# Metastable LaOCl*_x_* Phase Stabilization as an Effective Strategy for Controllable Chlorination of Ethane into 1,2-Dichloroethane

**DOI:** 10.3390/molecules30081746

**Published:** 2025-04-14

**Authors:** Yuting Li, Zihan Zhu, Xia Wu, Lei Ma, Xiaohui Sun, Qinggang Liu

**Affiliations:** 1 State Key Laboratory of Catalysis, Dalian Institute of Chemical Physics, Chinese Academy of Sciences, Dalian 116023, China; 2 University of Chinese Academy of Sciences, Beijing 100049, China; 3 Chemical Engineering and Resource Utilization, Northeast Forestry University, Harbin 150040, China; 4 State Key Laboratory of Heavy Oil Processing, China University of Petroleum (Beijing), Beijing 102249, China

**Keywords:** ethane chlorination, 1,2-dichloroethane, structural evolution, rare-earth oxychloride

## Abstract

LaOCl-mediated ethane chlorination into 1,2-dichloroethane offers a promising pathway for low-temperature, large-scale ethane upgrading. However, under Cl_2_-rich conditions, LaOCl undergoes detrimental chlorination into lanthanum chloride (LaCl_3_), accompanied by extensive surface hydroxylation. Such severe structural evolution limits the practical application of La-based catalysts under industrially relevant conditions. In this study, an alumina-stabilized La catalyst was prepared via a coprecipitation method. We demonstrated that strong La-O-Al interactions effectively resist structural degradation of La species under reaction conditions, enabling the modified catalyst to maintain exceptional stability under high Cl_2_ concentrations. At a C_2_H_6_/Cl_2_ ratio of 4:9, the optimized catalyst achieves an ethane conversion of 61%, with 1,2-dichloroethane selectivity sustained above 74% for 12 h without noticeable deactivation. In contrast, the bulk LaOCl counterpart suffers from rapid over-chlorination, shifting product dominance to trichloroethane within 10 h. Advanced spectroscopy characterization reveals that selectivity loss in LaOCl originates from phase collapse into LaCl_3_, whereas Al_2_O_3_ stabilization preserves the metastable LaOCl*_x_* phase in a highly dispersed state, ensuring selective C–Cl bond formation. These results highlight the critical role of stabilizing metastable oxychloride phases through robust metal oxide interactions, establishing a design framework for rare-earth catalysts in high-concentration chlorine environments.

## 1. Introduction

Over the past few decades, the direct conversion of ethane (C_2_H_6_) into value-added chemicals has emerged as a prominent research focus in heterogeneous catalysis [1,2,3,4,5], driven by its potential to revolutionize chemical feedstock supply through the utilization of abundant natural gas resources [6,7,8,9]. However, conventional C_2_H_6_ conversion processes are inherently constrained by thermodynamic limitations, with activation barriers persisting even at elevated temperatures approaching 700 K [10,11,12,13]. These harsh operational conditions typically induce undesirable side reactions, including coking and catalyst deactivation [14,15], while simultaneously incurring substantial energy requirements [16,17,18]. This fundamental challenge highlights the critical need for developing innovative catalytic systems that enable efficient ethane transformation under mild operating conditions.

Recently, Pérez-Ramírez and colleagues pioneered a catalytic process for the selective synthesis of 1,2-dichloroethane (1,2-C_2_H_4_Cl_2_) via ethane chlorination under mild conditions [19]. This strategy unlocks new opportunities for large-scale ethane updating, as 1,2-C_2_H_4_Cl_2_ serves as a pivotal precursor for polyvinyl chloride (PVC) [20,21,22,23], a commodity chemical with an annual global demand of 50 million tons that currently relies entirely on coal-and petroleum-derived feedstocks [24,25,26,27,28,29]. Compared to transition metals, non-redox rare-earth oxychlorides exhibit exceptional structural stability under harsh oxychlorination conditions [25,30,31,32], which inherently arise from the thermodynamically unfavorable chlorination under the temperatures required for C_2_H_6_ upgrading. This unique property positions rare-earth oxychlorides as preferred catalysts for C_2_H_6_ chlorination [33]. However, unsaturated metal cations with unpaired electrons on the oxychloride surface, which is critical for activating chlorine-containing intermediates [34,35], inevitably undergo structural degradation into inert chlorides under Cl_2_-rich conditions [36,37,38]. For instance, the representative lanthanum oxychloride (LaOCl) catalyst, which exceeds 80% selectivity for 1,2-C_2_H_4_Cl_2_, undergoes structural evolution during reaction, with the final catalyst structure primarily composed of LaCl_3_ and only trace amounts of detectable LaOCl phases [37]. To mitigate selectivity loss from phase collapse, such catalysts must operate under Cl_2_-lean conditions and maintain C_2_H_6_ conversion rates below 20%. This severely limits their industrial applications. Notably, since Pérez-Ramírez’s discovery in 2021 [19], no breakthroughs have been achieved in the field, highlighting the significant technical challenges that remain in converting C_2_H_6_ into target chlorinated hydrocarbons.

In this work, we demonstrate that robust La-O-Al interactions can effectively resist the segregation of La species in harsh Cl_2_ environments. X-Ray Diffraction (XRD) and X-ray photoelectron spectroscopy (XPS) analyses reveal that at 8.8% La loading, the LaOCl_x_ phase is well stabilized by Al_2_O_3_. The highly dispersed LaOCl*_x_* species, serving as active centers, can maintain the long-term controlled chlorination of C_2_H_6_. This optimized catalyst achieves 61% C_2_H_6_ conversion with >70% selectivity toward 1,2-C_2_H_4_Cl_2_ at a C_2_H_6_/Cl_2_ molar ratio of 4:9, maintaining stability for 12 h without deactivation. In contrast, we observed an obvious structural degradation of La catalytic centers in the bulk LaOCl counterparts. Intriguingly, this structural modulation directly governs the C_2_H_6_ chlorination pathway, shifting product selectivity from 1,2-C_2_H_4_Cl_2_ to trichloroethane (C_2_H_3_Cl_3_). This work elucidates the unique catalytic behavior of alumina-confined La-based catalysts, highlighting the potential of oxide-supported La single-atom catalysts for ethane chlorination.

## 2. Results and Discussion

**Figure 1:**
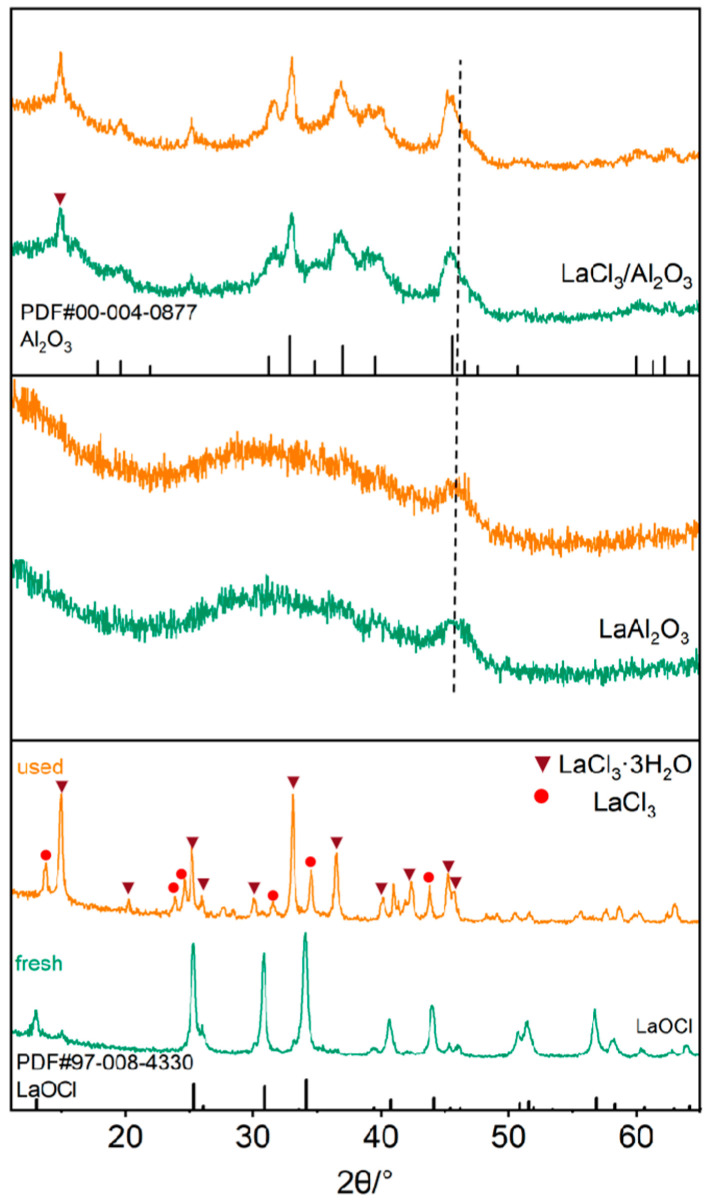
XRD patterns of selected catalysts prior to (green) and after (orange) ethane chlorination.

The Al_2_O_3_-stabilized La-based catalyst was synthesized via coprecipitation of La and Al nitrate precursors using ammonia as the precipitating agent, followed by calcination at 900 °C. Inductively coupled plasma optical emission spectroscopy (ICP-OES) analysis confirmed a La loading of 8.8 wt% in the catalyst (denoted as LaAl_2_O_3_). Previous studies by Pérez-Ramírez et al. have identified LaOCl as the primary active phase for C_2_H_6_ chlorination [19]. Accordingly, we prepared La_2_O_3_ via the precipitation method and exposed it to the feed gas atmosphere (C_2_H_6_:Cl_2_ = 4:9) for approximately 55 min to generate the LaOCl phase (Figure 1). For comparison, LaCl_3_ was loaded onto Al_2_O_3_ via impregnation to simulate surface over-chlorination to the LaCl_3_ phase, achieving a La loading of 9.0 wt% as determined by ICP-OES (labeled as LaCl_3_/Al_2_O_3_). Systematic characterization and catalytic performance of LaOCl, LaAl_2_O_3_, and LaCl_3_/Al_2_O_3_ were conducted to elucidate the structure-performance relationships of La-based catalysts in C_2_H_6_ chlorination.

XRD analysis was employed first to investigate the structural evolution of three La-based catalysts before and after C_2_H_6_ chlorination. As shown in Figure 1, the fresh LaOCl catalyst exhibited diffraction peaks matching the tetragonal LaOCl reference (ICCD 08-4330) [39]. After reaction, however, the LaOCl peaks significantly diminished, accompanied by the emergence of LaCl_3_ (ICCD 02-3146) and LaCl_3_·3H_2_O phases (ICCD 07-5266), indicating irreversible chlorination and lattice collapse under Cl_2_-rich conditions. In contrast, the LaAl_2_O_3_ catalyst demonstrated exceptional structural stability, with nearly identical XRD patterns before and after reaction. Its diffraction peak exclusively displayed Al_2_O_3_ peaks (ICCD 04-0877) with no detectable lanthanum oxide phases, suggesting uniform La incorporation into the alumina lattice. This hypothesis is further supported by a distinct shift of the Al_2_O_3_ (45°) peak to 46°—a consequence of lattice distortion induced by La doping [40,41]. Notably, the robust La-O-Al bonds formed during high-temperature calcination (900 °C) effectively stabilized La species, preventing LaCl_3_ formation even after 12 h of exposure to reactive atmospheres at 260 °C. For LaCl_3_/Al_2_O_3_, XRD patterns revealed Al_2_O_3_ (ICCD 04-0877) and LaCl_3_·3H_2_O (ICCD 07-5266) phases, signifying surface aggregation of LaCl_3_ due to its high loading.

**Figure 2:**
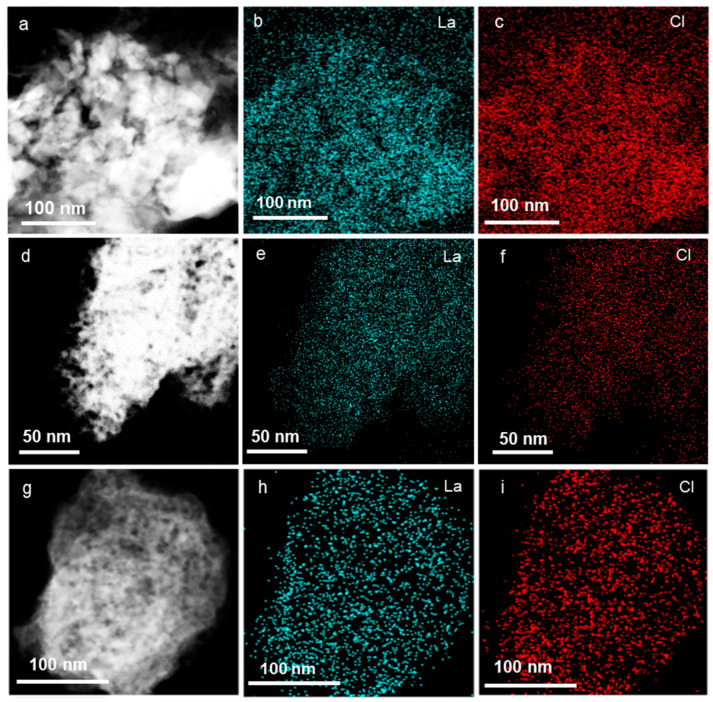
Elemental maps of catalysts after ethane chlorination. (**a**–**c**) LaOCl. (**d**–**f**) LaAl_2_O_3_. (**g**–**i**) LaCl_3_/Al_2_O_3_.

To further elucidate the structural evolution of LaOCl, LaAl_2_O_3_, and LaCl_3_/Al_2_O_3_ catalysts under reactive atmospheres, we analyzed their surface elemental distributions using energy-dispersive X-ray spectroscopy mapping (Figure 2). For the spent LaOCl (Figure 2a–c), Cl exhibited localized enrichment with spatial overlap between La and Cl, confirming irreversible chlorination of LaOCl into LaCl_3_. In contrast, LaAl_2_O_3_ (Figure 2d–f) showed negligible Cl signals and homogeneous distributions of La, Al, and O at the nanoscale, indicating that the robust La-O-Al interface suppressed Cl^−^ penetration and over-chlorination. The LaCl_3_/Al_2_O_3_ catalyst (Figure 2g–i) displayed uniform distribution of Cl on Al_2_O_3_, with Cl enrichment zones colocalized with La aggregates, consistent with XRD observations. These results demonstrate the extreme sensitivity of LaOCl to HCl-induced structural collapse and LaCl_3_ aggregation. Conversely, the La-O-Al interface in LaAl_2_O_3_ created a chlorine-resistant architecture that stabilizes LaOCl*_x_* species, providing an ideal active surface for controlled ethane chlorination.

**Figure 3:**
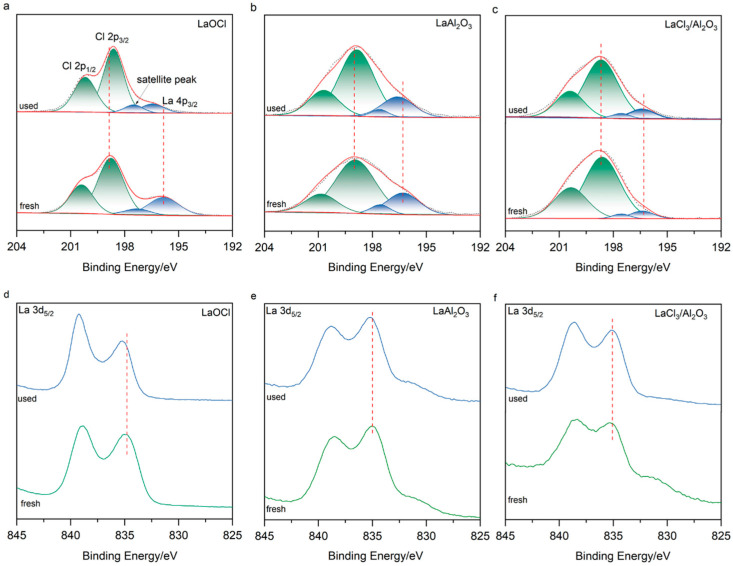
(**a**–**c**) Cl 2p & La4p_3/2_, (**d**–**f**) La 3d_5/2_ core level XPS spectra of fresh and spent catalysts.

XPS was conducted to elucidate the evolution of surface electronic states in LaOCl, LaAl_2_O_3_, and LaCl_3_/Al_2_O_3_ catalysts before and after reaction. As shown in Figure 3a–c, the Cl 2p spectra (193–203 eV) were deconvoluted into four characteristic peaks: spin-orbit splitting peaks of Cl^−^ species at 198.8 eV (Cl 2p_1/2_) and 200.4 eV (Cl 2p_3/2_), along with the La 4p_3/2_ peak at 195.8 eV and its satellite peak at 197.5 eV [42,43]. Systematic analysis of binding energy shifts and peak area ratios revealed distinct chlorination mechanisms. For the spent LaOCl catalyst (Figure 3a), the Cl 2p peaks shifted to lower binding energies (Δ ≈ 0.2 eV), while the La 4p_3/2_ and La 3d_5/2_ peaks shifted to higher binding energies (Δ ≈ 0.6 eV; Figure 3d). This confirmed electron transfer from La to Cl. A significant increase in the Cl 2p/La 4p_3/2_ peak area ratio further indicated surface over-chlorination. These observations suggest that continuous Cl coordination at La sites reduces the electron density of La centers, rationalizing the La 4p_3/2_ upshift. In contrast, the LaAl_2_O_3_ catalyst exhibited exceptional stability (Figure 3b,e). Its Cl 2p, La 4p_3/2_, and La 3d peaks showed minimal shifts (Δ ≤ 0.2 eV), and the Cl 2p/La 4p_3/2_ peak area ratio remained unchanged after reaction. This demonstrates that robust La-O-Al bonds stabilize surface La species in a LaOCl*_x_* configuration, preventing excessive chlorination under harsh conditions. Notably, LaCl_3_/Al_2_O_3_ showed invariant peak positions and Cl 2p/La 4p_3/2_ peak area ratios (Figure 3c,f), confirming structural integrity without significant surface reconstruction.

**Figure 4:**
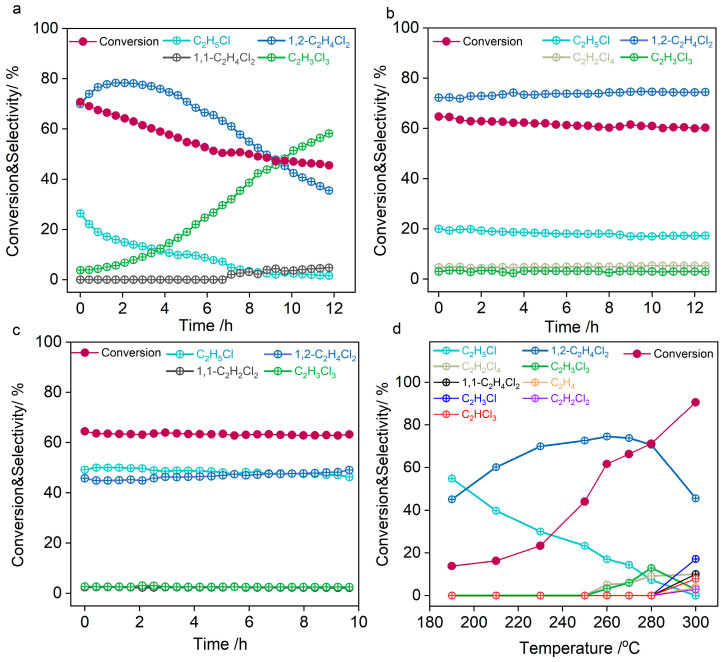
C_2_H_6_ conversion and product selectivities versus time-on-stream in ethane chlorination over (**a**) LaOCl, (**b**) LaAl_2_O_3_, and (**c**) LaCl_3_/Al_2_O_3_. (**d**) Conversion and selectivity of ethane chlorination over the LaAl_2_O_3_ catalysts under varying reaction temperatures. Reaction conditions: (**a**–**c**) C_2_H_6_/Cl_2_/N_2_ = 4:9:87, 260 °C, WHSV = 2000 mL·h^−1^·g^−1^. (**d**) C_2_H_6_/Cl_2_/N_2_ = 4:9:87, 190–300 °C, WHSV = 2000 mL·h^−1^·g^−1^.

The distinct structural evolution behaviors of LaOCl, LaAl_2_O_3_, and LaCl_3_/Al_2_O_3_ under reaction atmospheres result in differences in their catalytic activities. Through comparative analyses of product distributions and time-dependent evolution patterns in ethane chlorination over these three lanthanum-based catalysts, we elucidated the structure-dependent mechanism governing chlorination pathways. As illustrated in Figure 4a, LaOCl initially demonstrated superior catalytic activity with 70% ethane conversion, predominantly yielding 1,2-C_2_H_4_Cl_2_. This observation aligns with the findings of Pérez-Ramírez regarding LaOCl’s low activation energy and high catalytic efficiency in ethane chlorination [19]. However, a prolonged reaction duration induced the structural degradation of LaOCl, leading to the progressive accumulation of over-chlorinated products. After 10 h of operation, C_2_H_3_Cl_3_ became the dominant product (>50% selectivity), accompanied by a sharp conversion decline to 46%. The significant decline in ethane conversion rate was attributed to the over-chlorination of 1,2-C_2_H_4_Cl_2_, which consumes chlorine from the reactants and reduces the concentration of gas-phase Cl radicals required to drive the ethane conversion. The over-chlorination of 1,2-C_2_H_4_Cl_2_ may stem from the hydroxylation of LaOCl during surface chlorination. The hydrogen-bonding network formed by surface hydroxyl groups could enhance 1,2-C_2_H_4_Cl_2_ adsorption beyond optimal levels. Notably, in their study of La*_x_*Eu_1-*x*_OCl solid solutions, Weckhuysen et al. demonstrated that La^3+^ is readily chlorinated and acts as a chlorine buffer that can transfer chlorine to the active Eu^3+^ phase, thereby enhancing the methane oxychlorination activity [44]. This observation implies that chlorine atoms adsorbed on La^3+^ centers exhibit dynamic adsorption behavior. Under reactive atmospheres, the strong electrophilicity of La^3+^ centers may enable the adsorption of chlorine radicals beyond stoichiometric proportions, creating localized Cl radical enrichment. These chlorine reservoirs may serve as critical drivers in promoting the over-chlorination of 1,2-C_2_H_4_Cl_2_.

Intriguingly, the LaAl_2_O_3_ catalyst demonstrated remarkable stability under the reaction conditions of 260 °C and a C_2_H_6_/Cl_2_ ratio of 4:9. Throughout the 12-h reaction period, LaAl_2_O_3_ maintained consistent selectivity (~74%) for 1,2-C_2_H_4_Cl_2_ synthesis, with only a marginal decline in ethane conversion from 64% to 61% (Figure 4b). This performance stability is attributed to the robust La-O-Al interfacial structure. The LaOCl*_x_* species induced under reactive atmospheres efficiently catalyzed selective 1,2-C_2_H_4_Cl_2_ synthesis, offering theoretical guidance for designing stable C_2_H_6_ chlorination catalysts. XRD and XPS characterizations confirmed the structural stability of Al_2_O_3_-supported LaCl_3_ under reaction conditions, consistent with its stable catalytic performance: over a 10-h test, C_2_H_6_ conversion remained at 63% with 50% selectivity toward 1,2-C_2_H_4_Cl_2_ (Figure 4c). This phenomenon suggests that highly dispersed LaCl_3_ species stabilized by interfacial oxygen exhibit structural features like LaOCl*_x_*, enabling selective 1,2-C_2_H_4_Cl_2_ production. Notably, however, LaCl_3_ aggregation into inert crystalline grains on Al_2_O_3_ reduced chlorination capacity, resulting in 40% selectivity toward C_2_H_5_Cl. The residual catalytic activity of LaCl_3_/Al_2_O_3_ may originate from a minor fraction of highly dispersed LaCl_3_ species. These dispersed species, under the modulation of interfacial oxygen, may exhibit LaOCl*_x_*-like characteristics capable of stabilizing chlorine radicals. This inference can be confirmed by the study from Chen et al., which has shown that CuCl_2_ undergoes dissociative adsorption on an γ-Al_2_O_3_ (110) surface, where only one chloride ion binds to copper, and the other binds to the Al_2_O_3_ surface [45]. These findings collectively highlight that the formation of surface LaOCl*_x_* structures governs chlorination selectivity.

Through comprehensive analysis of temperature-dependent catalytic performance in ethane chlorination over LaAl_2_O_3_ systems, our study highlights the pivotal role of thermal effects in governing reaction pathway selection (Figure 4d). Notably, when reaction temperatures exceeded 300 °C, we observed the emergence of a competing dehydrochlorination pathway, producing ethylene (C_2_H_4_) and vinyl chloride (C_2_H_3_Cl). This phenomenon presents a significant challenge for developing efficient catalytic systems for selective chlorination of ethane to 1,2-C_2_H_4_Cl_2_, as the formation of unsaturated byproducts not only reduces target product yield, but also accelerates catalyst poisoning through coking mechanisms. The findings emphasize the importance of precise thermal management in chlorination processes to optimize reaction kinetics while suppressing dehydrogenation pathways. It is crucial to maintain reaction temperatures below 300 °C and implement strategies for selective intermediate stabilization.

**Figure 5:**
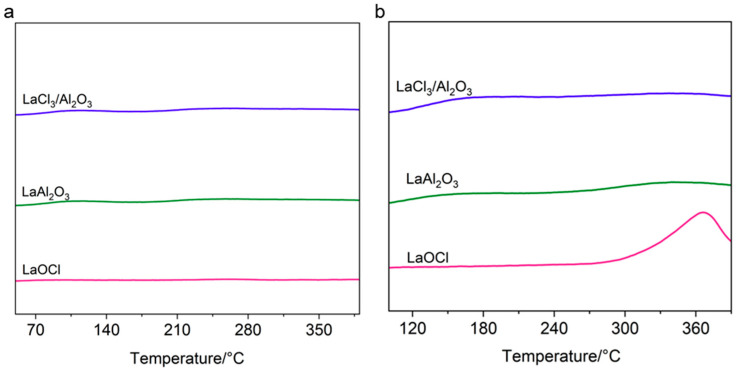
(**a**) C_2_H_5_Cl-TPD and (**b**) 1,2-C_2_H_4_Cl_2_-TPD of the spent catalysts.

To investigate the adsorption behavior of La-based catalysts toward intermediate species in the chlorination reaction, we conducted temperature-programmed desorption (TPD) experiments with C_2_H_5_Cl and 1,2-C_2_H_4_Cl_2_ on the spent LaOCl, LaAl_2_O_3_, and LaCl_3_/Al_2_O_3_ catalysts (Figure 5). C_2_H_5_Cl-TPD profiles (Figure 5a) revealed negligible desorption peaks (50–400 °C) across all catalysts, confirming their extremely weak adsorption capacity for C_2_H_5_Cl. This suggests that the adsorption of C_2_H_5_Cl on the catalysts is not a critical factor influencing its further chlorination to 1,2-C_2_H_4_Cl_2_. In the 1,2-C_2_H_4_Cl_2_-TPD tests (Figure 5b), the LaOCl catalyst exhibited a pronounced desorption peak at 360 °C, far exceeding the desorption peaks of LaAl_2_O_3_ and LaCl_3_/Al_2_O_3_ (Figure 5b). This indicates that surface chlorination of LaOCl during the reaction induces strong adsorption of 1,2-C_2_H_4_Cl_2_, leading to its over-chlorination to form C_2_H_3_Cl_3_. The absence of dichloroethane desorption peaks for LaAl_2_O_3_, and LaCl_3_/Al_2_O_3_ implies that neither LaOCl*_x_* nor LaCl_3_ species promote strong adsorption of 1,2-C_2_H_4_Cl_2_.

**Figure 6:**
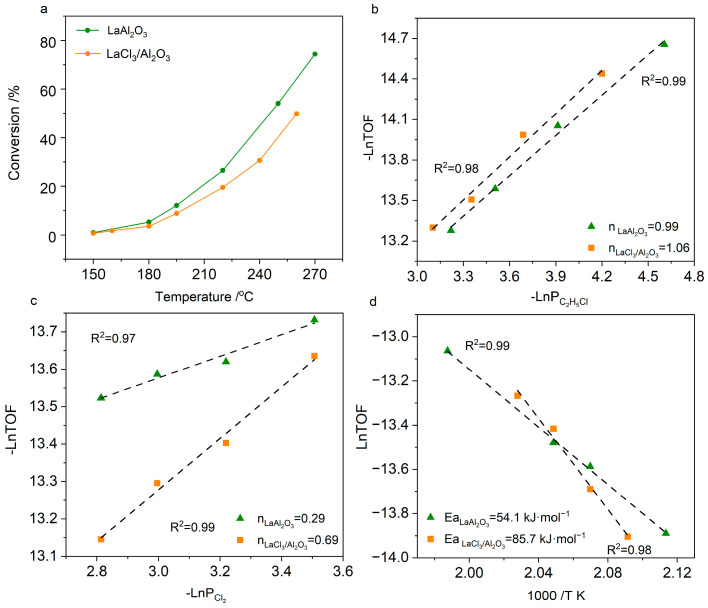
(**a**) Conversion as a function of temperature in the chlorination of C_2_H_5_Cl over the catalysts. Reaction conditions: C_2_H_5_Cl/Cl_2_/N_2_ = 3.5:5:91.5, 150–260 °C, WHSV= 5500 mL·h^−1^·g^−1^. (**b**) Reaction orders of C_2_H_5_Cl. (**c**) Reaction orders of Cl_2_. (**d**) Apparent activation energy of C_2_H_5_Cl chlorination. Reaction conditions (**b**–**d**): C_2_H_5_Cl:Cl_2_ = 1–5:2–5, 200–230 °C, WHSV = 5000–6000 mL·h^−1^·g^−1^.

Since ethane-to-chloroethane conversion occurs via gas-phase chlorine radical pathway independent of catalysts [46,47], the chlorination kinetics of C_2_H_5_Cl were investigated to elucidate structural effects. Steady-state kinetic tests and Arrhenius analysis systematically compared LaAl_2_O_3_ and LaCl_3_/Al_2_O_3_ in terms of light-off temperature, reaction orders, and apparent activation energy. As shown in Figure 6, LaAl_2_O_3_ exhibited superior low-temperature activity, with a 15 °C lower light-off temperature than LaCl_3_/Al_2_O_3_, confirming its enhanced catalytic capability. Notably, over the LaAl_2_O_3_ catalyst, the primary product was 1,2-C_2_H_4_Cl_2_, with byproducts mainly consisting of C_2_H_3_Cl_3_ and C_2_H_2_Cl_4_. Furthermore, no 1,1-C_2_H_4_Cl_2_ isomers were detected in the products, indicating that the chlorination of C_2_H_5_Cl primarily occurred on the catalyst surface. Conversely, aggregated LaCl_3_ clusters on Al_2_O_3_ hindered chlorination reaction, leaving unreacted C_2_H_5_Cl as the dominant byproduct. These performance disparities highlight that constructing La-O-Al interfaces optimizes active site dispersion, enhancing surface reactivity and selectivity.

Kinetic studies revealed near-first-order dependence on C_2_H_5_Cl for both catalysts (1.06 vs. 0.99, Figure 6b). Combined with the TPD characterization, it is evident that the reaction rate was not influenced by the strength of C_2_H_5_Cl adsorption. Worth noting is the significant difference in the reaction orders of Cl_2_ between the two catalysts. As shown in Figure 6c, the reaction order for Cl_2_ was 0.29 for LaAl_2_O_3_ and 0.69 for LaCl_3_/Al_2_O_3_, indicating that LaAl_2_O_3_ promotes Cl_2_ adsorption and activation. Combining the kinetic data with the catalytic performance, it can be conclusively inferred that the LaAl_2_O_3_ catalyst possessed more efficient chlorine adsorption sites, significantly accelerating the surface chlorination process. Notably, when the LaCl_3_ species on the catalyst surface was overly aggregated, this structural advantage was compromised, leading to the active sites being covered and the efficiency of chlorine activation being reduced. As shown in Figure 6d, the apparent activation energy for LaAl_2_O_3_ was 54.1 kJ·mol^−1^, significantly lower than the 85.7 kJ·mol^−1^ observed for the LaCl_3_/Al_2_O_3_ catalyst. This dynamic parameter difference was highly consistent with the catalytic performance evaluation and reaction order measurements.

**Figure 7:**
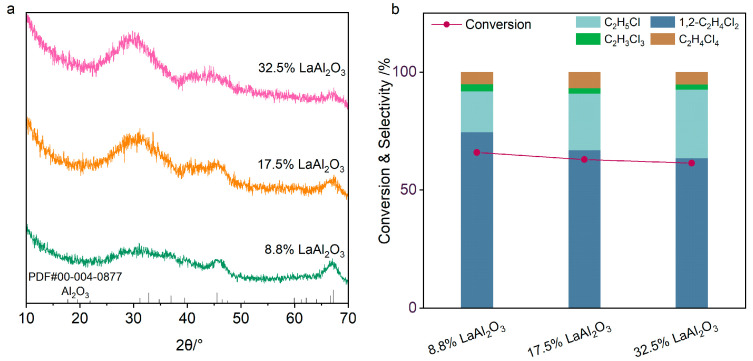
(**a**) XRD patterns of LaAl_2_O_3_ catalysts with La loadings of 8.8%, 17.5% and 32.5%. (**b**) Conversion and selectivity of ethane chlorination over the LaAl_2_O_3_ catalysts. Reaction conditions: C_2_H_6_/Cl_2_/N_2_ = 4:9:87, 260 °C, WHSV = 2000 mL·h^−1^·g^−1^.

The exceptional ethane chlorination performance of LaAl_2_O_3_ catalysts originates from their structural stability, prompting us to investigate the maximum La content that Al_2_O_3_ can stabilize to resist surface chlorination under reactive conditions. To address this, we engineered LaAl_2_O_3_ catalysts with elevated La loadings of 17.5% and 32.5%. XRD analysis (Figure 7a) revealed highly dispersed La species within the alumina lattice, as indicated by significant peak broadening at 46° and 67° corresponding to γ-Al_2_O_3_. Nevertheless, excessive La/Al ratios induced partial structural instability under chlorination atmospheres, resulting in LaCl_3_ formation and a marked reduction in 1,2-C_2_H_4_Cl_2_ selectivity (Figure 7b). These results unequivocally demonstrate that, while Al_2_O_3_ serves as an effective stabilization platform for La species, precise control over La loading is imperative to optimize both structural resilience and catalytic efficiency.

**Figure 8:**
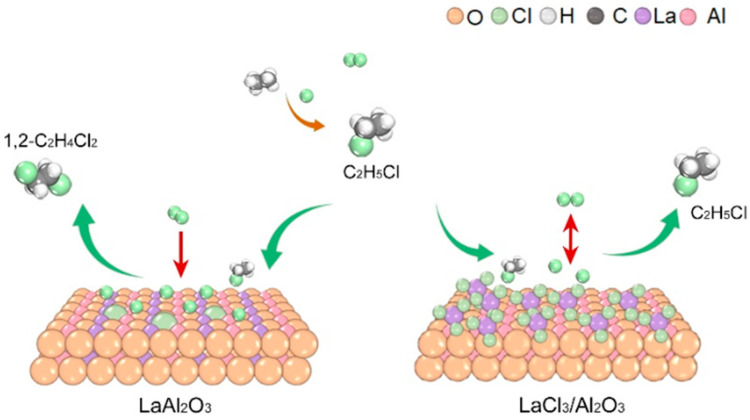
Schematic illustration for the mechanism of ethane chlorination on LaAl_2_O_3_ and LaCl_3_/Al_2_O_3_.

Based on the experimental analyses above, we present a mechanistic model for ethane chlorination (Figure 8). The catalyst-independent C_2_H_6_ conversion observed in Figure 4 suggests that the initial chlorination step to C_2_H_5_Cl is primarily mediated by gas-phase chlorine radicals. Weak adsorption of C_2_H_5_Cl, as evidenced by C_2_H_5_Cl-TPD profiles and its high reaction order across all catalysts, indicates that C_2_H_5_Cl adsorption/activation is not the rate-determining step (RDS) for its subsequent conversion to 1,2-C_2_H_4_Cl_2_. The marked disparity in Cl_2_ reaction orders between LaAl_2_O_3_ and LaCl_3_/Al_2_O_3_ catalysts highlights that Cl_2_ activation and the stabilization of chlorine radicals on the catalyst surface likely govern the RDS for C_2_H_5_Cl chlorination. In LaAl_2_O_3_, LaOCl*_x_* centers stabilized by the La-O-Al framework act as electrophilic sites to efficiently anchor chlorine radicals. In contrast, LaCl_3_, with its saturated Cl coordination, impedes chlorine radical access, thereby suppressing further chlorination. These findings underscore the critical importance of stabilizing La in a LaOCl*_x_* configuration rather than a fully chlorinated state (LaCl_3_), which mechanistically explains the experimentally observed high selectivity for C_2_H_6_-to-1,2-C_2_H_4_Cl_2_ conversion on LaAl_2_O_3_ surfaces.

## 3. Experimental Section

### 3.1. Catalyst Preparation

Lanthanum nitrate hexahydrate (La(NO_3_)_3_·6H_2_O), lanthanum chloride heptahydrate (LaCl_3_·7H_2_O), and aluminum nitrate nonahydrate (Al(NO_3_)_3_·9H_2_O) were purchased from Macklin Chemical Reagent Factory. Ammonia (NH_3_·H_2_O) was purchased from Tianjin Damao Chemical Reagent Factory.

La_2_O_3_ was synthesized via the ammonia precipitation method. Initially, 5.6 g La(NO_3_)_3_·6H_2_O was dissolved in 150 mL of deionized water. An alkaline solution was prepared by dissolving 20 mL NH_3_·H_2_O with a mass fraction of 25 vol% in 150 mL of deionized water. This alkaline ammonium hydroxide solution was then gradually added to the acidic La(NO_3_)_3_ solution while stirring continuously. The resulting mixture was placed on a magnetic stirrer and stirred for 8 h until the pH value of the solution exceeded 8. After that, the mixture was subjected to centrifugation to separate the solid phase, followed by drying and milling to obtain a fine powder. Finally, the powder was calcined at a high temperature of 800 °C for 3 h, yielding the La_2_O_3_ catalyst with a well-defined crystalline structure.

The synthesis of LaAl_2_O_3_ was carried out using the ammonia precipitation technique. To begin with, 0.52 g La(NO_3_)_3_·6H_2_O and 11.38 g Al(NO_3_)_3_·9H_2_O (17.5% LaAl_2_O_3_: 1.13 g La(NO_3_)_3_·6H_2_O and 10.93 g Al (NO_3_)_3_·9H_2_O, 32.5% LaAl_2_O_3_: 1.69 g La(NO_3_)_3_·6H_2_O and 10.52 g Al (NO_3_)_3_·9H_2_O). Meanwhile, an alkaline solution was prepared by dissolving 25 mL NH_3_·H_2_O with a mass fraction of 25 vol% in 150 mL of deionized water. This alkaline ammonium hydroxide solution was subsequently introduced into the acidic solution containing La(NO_3_)_3_ and Al(NO_3_)_3_ with continuous stirring. The resultant mixture was placed onto a magnetic stirrer and stirred for 8 h until the pH of the solution exceeded 8. Following this, the mixture underwent centrifugation to separate the solid phase, which was then dried and milled. Lastly, the milled material was calcined at a high temperature of 900 °C for 3 h, resulting in the formation of the LaAl_2_O_3_ catalyst. Prior to catalytic evaluation, LaAl_2_O_3_ was activated by treatment in a feed mixture containing HCl/N_2_ = 10:90 at 260 °C for 6 h.

The alumina (Al_2_O_3_) carrier was prepared by the co-precipitation method. Initially, Al(NO_3_)_3_·9H_2_O was dissolved in deionized water with continuous stirring until a clear solution was formed. Subsequently, NH_3_·H_2_O was slowly added while stirring until the solution pH reached around 8–9 (monitored using pH paper). At this stage, a white flocculent precipitate of aluminum hydroxide (Al(OH)_3_) was observed. The solution was then allowed to stand for 1–2 h to age the precipitate and improve its crystallinity. After centrifugation, drying, and milling, the Al_2_O_3_ was obtained by calcination at 900 °C for 3 h. The LaCl_3_/Al_2_O_3_ catalyst was synthesized via the incipient wetness impregnation method. Initially, 0.37 g LaCl_3_·7H_2_O was dissolved in water to prepare a solution. One gram of the previously prepared alumina was impregnated with a specific amount of the LaCl_3_ solution, ensuring that the volume of the impregnation solution matched the volume of liquid absorbed by 1 g of alumina. The impregnated alumina was then placed in an oven at 155 °C and dried for 4 h to yield the LaCl_3_/Al_2_O_3_ catalyst.

### 3.2. Characterization

High-resolution transmission electron microscopy (HRTEM) images and energy-dispersive X-ray spectroscopy (EDS) analyses were performed using a JEM2100F microscope (JEOL Ltd., Tokyo, Japan), which was operated at an accelerating voltage of 200 kV. The powder X-ray diffraction (XRD) patterns were obtained using a PW3040/60 X’Pert ProSuper diffractometer (PANalytical, Malvern, UK), which was equipped with a Cu Kα radiation source. The instrument was operated at a voltage of 40 kV and a current of 40 mA. X-ray photoelectron spectroscopy (XPS) was measured on a Thermofisher ESCALAB 250Xi instrument (Thermo Fisher Scientific, Waltham, MA, USA), which applied monochromatic Al Kα radiation (hυ = 1486.6 eV) as the X-ray source. The concentration of La in the samples was determined using an inductively coupled plasma optical emission spectrometer (ICP-OES). C_2_H_5_Cl-TPD and 1,2-C_2_H_4_Cl_2_-TPD experiments were carried out using a Micromeritics AutoChem II 2920 chemisorption analyzer (Micromeritics, Norcross, GA, USA). Prior to the measurements, the sample was pretreated by drying in a helium (He) flow at 250 °C for 150 min. It was then cooled down to 40 °C and exposed to C_2_H_5_Cl (or 1,2-C_2_H_4_Cl_2_) for 60 min to allow adsorption. Following this, the gas was switched to He for purging, and the temperature was increased at a rate of 10 °C per minute until it reached 400 °C. Throughout the process, a thermal conductivity detector was employed to monitor and record the signals.

### 3.3. Catalyst Evaluation

Ethane chlorination (EC) was executed at atmospheric pressure in a self-made continuous-flow fixed-bed reactor. The gases C_2_H_6_ (8% in N_2_), C_2_H_5_Cl (9% in N_2_), Cl_2_ (18% in N_2_), Ar (carrier gas), and N_2_ (carrier gas) were fed into a mixing unit at controlled flow rates using digital mass-flow controllers (Bronkhorst^®^, Veenendaa, The Netherlands). Catalytic performance was evaluated in a fixed-bed quartz reactor with an inner diameter of 8 mm. The inlet feed gas mixture consisted of C_2_H_6_/Cl_2_/N_2_ in a ratio of 4:9:87. The reaction was conducted at a temperature of 260 °C, with a total gas flow rate of 16.67 mL/min. A catalyst loading of 0.5 g was used, resulting in a weight hourly space velocity (WHSV) of 2000 mL·h^−1^·g^−^^1^. A gas chromatograph (GC) equipped with a FID detector (PANNA, Changzhou, China) was used for online analysis of the feed gas and the reaction products.

Kinetic studies of the catalysts were conducted in a fixed-bed quartz reactor with an inner diameter of 8 mm. A 0.2 g portion of catalyst was loaded into the reactor and pretreated in a feed gas mixture (C_2_H_6_/Cl_2_/N_2_ = 4:9:87) at 260 °C for 10 h under atmospheric pressure. Subsequently, the reaction was performed with the reactor bed temperature varying from 140 °C to 260 °C. The feed composition was adjusted to C_2_H_5_Cl:Cl_2_ = 1–5:2–5 at temperatures between 200 and 230 °C. The catalyst loading remained at 0.2 g, corresponding to a weight hourly space velocity (WHSV) of 6000 mL·h^−^^1^·g^−^^1^. For each experimental condition, measurements were taken at 20-min intervals, and the average of at least three measurements was utilized to determine the concentrations.

C_2_H_6_ and C_2_H_5_Cl conversions were calculated from the following equation:

$${\mathrm{Con}}_{C_{2}H_{6}(C_{2}H_{5}\mathrm{Cl})}=\frac{C_{2}H_{6}{\left(C_{2}H_{5}\mathrm{Cl}\right)}_{\mathrm{inlet}}-C_{2}H_{6}{\left(C_{2}H_{5}\mathrm{Cl}\right)}_{\mathrm{outlet}}}{C_{2}H_{6}{\left(C_{2}H_{5}\mathrm{Cl}\right)}_{\mathrm{inlet}}}$$

The product selectivity X (x = C_2_H_4_, C_2_H_5_Cl, C_2_H_3_Cl, 1,2-C_2_H_4_Cl_2_, 1,1-C_2_H_4_Cl_2_, and 1,1,2-C_2_H_3_Cl_3_ selectivity) was calculated according to the following equation,

$${\mathrm{Sel}}_{x}=\frac{X_{\mathrm{outlet}}}{C_{2}H_{6(\mathrm{inlet})}-C_{2}H_{6(\mathrm{outlet})}}$$

where “inlet” and “outlet” represent chemicals in the inlet and outlet, separately.

The reaction order is typically determined experimentally using the rate expression

*r* = *k*⋅[*A*]^*m*^[*B*]^*n*^

where *r* is the reaction rate, *k* is the rate constant, and *m* and *n* represent the partial reaction orders with respect to reactants *A* and *B*, respectively, measuring the initial reaction rate under varying initial concentrations of one reactant while keeping others constant. The reaction order for a specific reactant is the slope derived from the logarithmic relationship of

ln *r* = ln k + *m*⋅ln [A] + *n*⋅ln [B]

The activation energy (*E_a_*) is calculated using the Arrhenius equation,

(1) $$k=A\cdot e^{-E_{a}/(RT)}$$

where *k* is the rate constant, *A* is the pre-exponential factor, *R* is the gas constant (8.314 J·mol^−1^·K^−1^), and *T* is the temperature in Kelvin. By measuring rate constants (*k*) at multiple temperatures, Ea can be determined from the linearized form:

$$lnk=lnA-\frac{E_{a}}{R}\cdot \frac{1}{T}$$

A plot of ln*k* versus 1/*T* (Arrhenius plot) yields a straight line with the slope (−*E_a_*/*R*), allowing *E_a_* to be derived.

In the real experiment, both reaction orders and activation energies were calculated based on the average turnover frequencies (TOF) obtained at low conversion levels. Since the same reactor configuration was employed, the active sites on the catalyst surface remained consistent across experiments, and a normalization procedure was consequently applied. Critically, this normalization does not alter the slope of the LnTOF vs. LnPCl_2_ relationship (i.e., the reaction order). The residence time used for calculating the average TOF was determined by the gas flow rate. Therefore, the data utilized for TOF calculations included the feed gas concentration, gas flow rate, and measured conversion. It is important to note that the gas flow rate was intentionally increased when necessary to ensure conversions remained below 20%.

## 4. Conclusions

This work systematically deciphers the structure-dependent selectivity and kinetics of La-based catalysts in ethane chlorination. LaOCl, due to its thermodynamic instability in a Cl_2_-rich environment, undergoes irreversible chlorination (LaOCl → LaCl_3_), enhancing its adsorption of 1,2-C_2_H_4_Cl_2_ and triggering the over-chlorination of C_2_H_6_. By confining La within robust Al_2_O_3_ and with the assistance of La-Al-O bonds, La can be stabilized in a LaOCl*_x_* structure, effectively promoting the selective generation of 1,2-C_2_H_4_Cl_2_. Using the LaCl_3_/Al_2_O_3_ model catalyst, we confirmed that aggregated LaCl_3_ particles, due to their weakened chlorine adsorption activation capability, severely limit the further chlorination of C_2_H_5_Cl. Through further kinetic experiments, we validated the critical role of LaOCl*_x_* sites in the selective chlorination of C_2_H_6_. Kinetic analysis confirmed that LaOCl*_x_* sites stabilized by La-Al-O interfaces reduced chlorination barriers to 54.1 kJ·mol^−1^, achieving high selectivity (74%) and long-term stability for 1,2-C_2_H_4_Cl_2_. These findings elucidate the unique catalytic behavior of oxide-confined La-based systems, highlighting the potential of single-atom La catalysts in C_2_H_6_ chlorination and providing a theoretical framework for designing efficient, stable industrial chlorination catalysts.

## Data Availability

The original contributions presented in this study are included in the article. Further inquiries can be directed to the corresponding authors.
